# A management system for randomized clinical trials: A novel way to supply medication

**DOI:** 10.1371/journal.pone.0212475

**Published:** 2019-02-22

**Authors:** Mauro Cortellini, Alice Casagrande, Giuseppe Fornaciari, Daniela Del Sette, Giuliana Gargano, Maria Gaetana Di Mauro, Gabriella Saibene, Eleonora Bruno, Anna Villarini, Elisabetta Venturelli, Franco Berrino, Ivan Baldassari, Andreina Oliverio, Patrizia Pasanisi

**Affiliations:** 1 Department of Preventive & Predictive Medicine, Epidemiology Unit, Foundation IRCCS National Cancer Institute of Milan, Milan, Italy; 2 Department of Nervous System and Behavioural Sciences, University of Pavia, Pavia, Italy; 3 SC Pharmacy, Foundation IRCCS National Cancer Institute of Milan, Milan, Italy; 4 Department of Biomedical Sciences for Health, University of Milan, Milan, Italy; Karolinska Institutet, SWEDEN

## Abstract

**Background:**

Randomized controlled clinical trials require management effort, involving huge organizational, economic and informatics investments. Information technology offers opportunities to approach clinical trial methodology in new ways. However, there are only a few reports of computerized data and drug management systems.

**Objective:**

This paper describes a novel software created specifically for the management of a randomized trial of diet and metformin in people with metabolic syndrome (the Me.Me.Me. trial).

**Methods:**

Me.Me.Me. is an ongoing phase III randomized controlled trial in healthy people with metabolic syndrome to test the hypothesis that comprehensive lifestyle changes and/or metformin can prevent age-related chronic non-communicable diseases. To manage all the phases of the trial, we created a software which is a state pattern machine, user friendly, web-based, able to maintain the correct balance between randomization groups, and structured in various levels of security in order to guarantee the participant’s privacy and compliance with the study protocol. The software achieves budget savings: drug management is not based on *patients’ packs*, but on the actual need for drugs according to each participant’s “state”, with strict guidelines for the handling and supply of medication.

**Results:**

The trial is ongoing and recruitment will close on August 31, 2018. To date, 11737 bottles of metformin/placebo have been dispensed to 1054 randomized participants, with drug savings of 29.5%.

**Conclusions:**

A software which takes into account the “state” of participant might be a powerful resource for developing and managing clinical trials, helping avoid poor treatment allocation, and wastage of drugs and money.

**Me.Me.Me. trial:**

EUDRACT no. 2012-005427-32.

ClinicalTrials.gov Identifier: NCT02960711.

## Introduction

Randomized controlled clinical trials require a substantial management effort, involving huge organizational, economic and informatics investments [1,2]. The management, transfer and storage of data generated by these studies can all create obstacles to their success. These include a lack of efficient and homogeneous coordination of study activities among study team members; lack of real-time data cleaning and reporting; lack of resources for data recording; and endless amounts of data that must be stored. Furthermore, clinical trials often require the sequential administration of drugs/placebo and the management of drugs supply is always a critical point.

Information technology offers new ways to approach clinical trial methodology. The efficiencies of web-based clinical data management systems could provide solutions to several of these obstacles. However, despite the importance of medication management in clinical trials, the scientific literature offers few reports of computerized drug management systems [3,4,5].

Here we describe an example of how a small research team, assisted by a limited IT group, can build a good-quality Clinical Trial Management System (CTMS) capable of adequate data and drug management without huge resources.

## Objective

This report describes a new way of approaching clinical trials.

We created an “*ad hoc*” software for the management of all the phases of a randomized controlled trial of Mediterranean diet and metformin in healthy people with metabolic syndrome (the Me.Me.Me. trial) (S1 and S2 Files) [6]. We are aware that several commercial software systems are already available for clinical trial management (e.g. Electronic Data Capture systems, EDC) [7]. However, our aim was to establish a new way to reinforce the relationship between the research team and the drug supplier. This software improves the current state of the art, offering a new approach to planning drug orders with the pharmaceutical company in order to avoid waste and save money. This is essential in a no-profit context where funding is usually a problem.

We describe a novel, flexible, home-made software to manage a clinical trial dynamically and cheaply.

## Methods

Ethical approval was obtained from the institutional review board and ethics committee of the Milan National Cancer Institute. (EUDRACT no. 2012-005427-32).

### Me.Me.Me. trial design (S1 Fig)

Me.Me.Me. stands for metabolic syndrome, Mediterranean diet and metformin [6]. This is an ongoing Phase III randomized controlled trial on people with metabolic syndrome, to test the hypothesis that a comprehensive dietary intervention with moderate calorie and protein restriction (including Mediterranean-macrobiotic recommendations and recipes) and/or treatment with metformin can prevent age-related chronic non-communicable diseases. Observational data have previously suggested the metformin treatment is associated with a lower incidence/mortality of several chronic diseases [8,9] including cancer [10], coronary heart disease [11], stroke [12], and diabetes [13].

The study design is 2x2 factorial with 1600 volunteers to be randomized in four groups of 400 each and allocated to the following treatments:

- metformin (1700 mg/day) [14] + active dietary intervention
- placebo + active dietary intervention
- metformin (1700 mg/day) alone
- placebo alone.

The trial is scheduled to last five years.

Me.Me.Me. is a monocentric trial with a single enrolment center at the Milan National Cancer Institute.

At baseline, according to the trial design (S1 Fig), participants are asked to sign an informed consent form and attend an anthropometric visit, giving blood and urine samples. The baseline measurements serve to check the presence of the metabolic syndrome and the absence of exclusion criteria. A participant is eligible if s/he has at least three of the following metabolic disorders: abdominal obesity (waist circumference 85 cm or more for women and 100 cm or more for men), hyperglycemia (100 mg/dL or more), hypertriglyceridemia (150 mg/dL or more or are receiving hypertriglyceridemia treatment), low high-density lipoprotein (HDL) (50 mg/dL or less for women and 40 mg/dL or less for men or are receiving treatment for hypercholesterolemia) and high blood pressure (130/85 mmHg or more or under treatment for high blood pressure).

The exclusion criteria are: diabetes, fasting glycemia more than 126 mg/dL in two repeated blood samples or concomitant treatment with metformin, diagnosis of cancer in the last five years (except skin carcinomas), serum creatinine more than 124 micromol/L, proteinuria, concomitant treatment with potassium-sparing diuretics or proton pump inhibitors, excessive alcohol consumption.

Once a year each participant repeats the anthropometric visit and blood sampling, on approximately the same date as the previous one for the five years of follow-up as requested by the study protocol (3.5-year follow-up on average).

According to the amendment n.1 to grant agreement 322752, the Me.Me.Me. trial obtained a time extension of one year and it will end by July 31, 2019. Recruitment will close on August 31 2018, and at the time of writing 1755 volunteers signed an informed consent.

### The Me.Me.Me. information software system

The Me.Me.Me. software requires Microsoft technology. The software has been designed and developed according to the specific needs of the study without using any commercial tool. All the informatic code was written by the software developer of the Fondazione IRCCS Istituto Nazionale dei Tumori di Milano.

The Me.Me.Me. software ensures a clear relationship among all the research figures involved in the trial. Our software requires the following pre-requisites:

Operative System: Microsoft Server WEB 2008 R2 64bit.

DataBase: Microsoft SQL Server 2012.

Programming: ASP.NET MVC4.

The software is:

- User-friendly: ease of use was a key goal in designing the software. In fact trial personnel do not need an informatics background. Our software enables them to handle all the procedures in complete autonomy. They are only asked to follow the step-by-step instructions that appear on the screen.
- Web-based. Each person involved in the trial has their own password and can access the database wherever they need: all the data are stored in our on-line software. This is an important user-friendly and web-based entry method for managing clinical data.
- A state pattern machine: each participant is a “state” and follows specific steps according to the study protocol and timesheet.
- Able to maintain the correct balance between randomization groups and safeguard the double-blind scheme, while minimizing drug waste and maximizing efficiency.
- Structured in various levels of security in order to guarantee the participant’s privacy and compliance with the study protocol in accordance with the Declaration of Helsinki [15] and the Ethics Guidelines for Clinical Research (GCP) [16].

### Randomization

The software balances the randomized groups [17] to make sure they are comparable at any time. The Me.Me.Me. trial (S1 Fig) comprises two distinct randomizations: DietRandomization first (based on registration data) and TreatmentRandomization (based on inclusion criteria data) (S1 Fig). This takes account of the fact that several weeks may pass between the date of recruitment and the date of the anthropometric visit and blood sampling.

The software balancing mechanisms are:

- Automatic
- Not modifiable by the operators
- Accessible to operators at any time by summarizing statistics.

### Diet randomization

Two features of the population are considered to balance the diet randomization:

- Sex: male/female
- Family relationship: members of the same family are included in the same randomization group.

The software assigns each participant to either the active lifestyle intervention or the no intervention arm when the data manager saves the registration data [18]. The assignment is recorded in the DietRandomization field.

Blue is used for the Active lifestyle intervention, and green for the control group. The data manager can check the correct balance of randomized groups at any time on the homepage (Table 1).

**Table 1:** Correct balancing of the groups. The term “Proband” refers to the first family member enrolled in the trial and “Familiar” refers to subsequent family members enrolled.

Diet Randomization						
	Blue		Green			
	Male	Female	Male	Female		Total
**Proband**	327	479	349	456		**1611**
**Familiar**	13	68	5	58		**144**
**Total**	**340**	**547**	**354**	**514**		**1755**

### Treatment randomization

Two features of the population are considered to balance treatment randomization:

- Sex: male/female
- Age: up to 67/over 67 years.

Treatment randomization starts only if the participant fulfills all the following entry criteria:

- Complete registration data on lifestyle, 24-h diet diary, physical activity diary, concomitant medications in the electronic Case Report Form (eCRF)
- Presence of the metabolic syndrome and absence of exclusion criteria (based on anthropometric measures, blood and urine samples)

and

- No adverse events in the first 30 days of metformin treatment (schedule: one 500 mg metformin tablet/day) (S1 Fig).

The data entry procedure unlocks the randomization for metformin/placebo and the software manages the correct balance among the groups. The TreatmentRandomization field is immediately visible in an encrypted form, using a double-blind scheme (“X” and “Y” instead of “metformin” or “placebo”). Then the software assigns the right drug bottle number for the first treatment according to the protocol flow chart (S1 Fig). For subsequent supplies, the data manager has only to follow the indications provided by the software.

### Medication management schedule

After randomization for treatment the software provides a supply to cover two months of treatment (62 tablets of metformin/placebo) according to the protocol (one 850 mg tablet/day of metformin/placebo) (S1 Fig). The next step is to supply a full treatment (62 tablets of metformin/ placebo), to cover one month (two tablets, for a total of 1700 mg/day of metformin/placebo). Then, all the participants are given a six-month full treatment supply. If anyone shows poor tolerance to metformin or regression of the metabolic disorder along the trial, the Principal Investigator (PI) can adjust the dosage (e.g. giving a half-dose). The Me.Me.Me. flexible software can manage all the participants’ dosage changes throughout the trial.

The software can block the progress of each participant’s “state” in case of non-compliance, withdrawal of consent or serious adverse events. All adverse events are monitored, collected and recorded in the eCRF along the trial.

At the beginning of the trial, an allocation list was generated involving a theoretical and a redundant number of bottles to randomize. About 200,000 bottle codes were generated. We used an allocation block design, which involves the construction of a random metformin/placebo sequence to maintain a balance between the two arms. The database-generated blocks comprised four bottles: two for metformin and two for placebo, in a random sequence (e.g. metformin-metformin-placebo-placebo, placebo-metformin-placebo-metformin). The software administrator has to give the drug manufacturer a copy of the not encrypted list and a second copy is entered in the database. The list sent to the supplier guarantees the anonymity of participants and treatment; it contains only instructions for packaging the bottles, according to Good Manufacturing Practice (GMP). Bottle codes and type of treatment are the sole information available to the supplier. If a researcher tries to access the “physical” database (where the trial data reside), s/he will not be able to go back to the participant's treatment and will see the screen shown in S2 Fig.

The software provides a medication management schedule for each participant. It shows an “allocation register” with all the dates for deliveries to each participant and expiry dates of the drugs.

### Warehouse management

Once the randomization arm has been defined, drug allocation is carried out time after time, for every single step for each participant (“state” of the participant). The virtual warehouse in which the drug/placebo are stored has been planned so as to save every single bottle of medication. Frequently, trials adopt *patients’ packs* for drug/placebo. This procedure means ordering a large amount of drug at the beginning of the trial, with a fixed assignment of full supplies to each participant. This can lead to misspending and mismanagement of the drugs because of:

- participants who drop out of the trial;
- the difficulty of compliance with the estimated accrual rate;
- the difficulty of compliance with the full dosage of the drugs
- expiry date of the drugs and delivery of batches.

We designed the software to achieve savings. Therefore, the assignment of bottles depends on needs, saving waste. The Me.Me.Me trial does not adopt *patients’ packs*, but follows each participant’s actual need of drugs according to his/her “state”. The flexible warehouse management is the real novelty of our software. In the management of a drug, warehouse optimization is vital. One must avoid shortages of drugs but the orders must be optimized to avoid wastage or returns. The link with the supplier and the supply agreement are essential.

### Requirements for suppliers and for expired drugs

The European legislation about GCP on Investigational Medical Products (IMP) was adopted by the Competent Authority in Italy in 2007 [19]. Art.8 specifies that hospital pharmacies can prepare and pack IMP only for trials on subjects with the features described in the marketing authorization. Primary prevention trials are often designed to test drug efficacy outside the regular marketing authorization–as we are using metformin not for type II diabetes but for persons with metabolic syndrome. In these cases the PI must sign a contract with the pharmaceutical companies that are legally able to prepare and pack the IMP.

Orders for supplies from the pharmaceutical company depend on: how many bottles are needed for already randomized participants (A); how many are needed for estimated future participants taking into account the trend of recruitment (B1); how many are needed for participants in the phase between baseline screening and randomization (who are receiving metformin, 500 mg day for the first 30 days (B2); estimated withdrawals (C). These variables are computed by the following formula: (A+B1+B2)–C.

The PI plans the drug orders. Within 30 days (established in the initial agreement), the supplier has to schedule a delivery of drugs with an expiry date of at least twelve months. The pharmaceutical company has changed the expiry date during the course of the trial as the stability tests in bottles were concluded. Therefore, along the study, the expiry date of drug has been extended from 12 to 48 months.

Once received, the medications are shipped and stored at the host institution’s pharmacy, for dispensing in accordance with software assignations. The procedure can be summarized as follows:

1. The software provides an estimate of the needs for the following 12 months according to the rate of recruitment, stocks held and the states of each participant;
2. The pharmaceutical company has to deliver the metformin/placebo within 60 days to the center’s pharmacy;
3. Upon receipt of the supply, the software manager registers the whole amount, batch number, date of loading and expiry date in the database.

Needs are estimated at least 30 days before the last batches expire. The order is promptly communicated to the pharmaceutical company by certified e-mail. The software indicates drugs that have expired and they are disposed of by the host pharmacy. The software does not allow the assignment of drugs close to the expiry date or that have expired.

### Breaking the blind

The software includes a procedure for breaking the blind which can be activated only in case of major medical conditions or hospital admission (emergency cases). The PI is allowed to break the blind so as to see which treatment the participant was assigned. S/he can promptly enter the database and clearly display the name of the participant and the treatment (metformin/placebo). The PI is the only person with an emergency role. The software records every single entry by the PI.

### Data security

The system security is divided up on various levels and concerns:

- web server security. The server location is: CLOUD server on web farm ARUBA Spa. Firewall and integrated security system based in the cloud which allows redundant server structures in cluster and replicated storages.
- access to the application currently in use at the Fondazione IRCCS Istituto Nazionale dei Tumori (www.progettomememe.nethttp://www.diana5.net/). The access is through personal ID passwords generated directly by the software with random criteria; they must be 18 characters long and must contain uppercase or lowercase characters and punctuation symbols. The software administrator can enter the server for maintenance purposes, using a dedicated mechanism. He connects to the Remote Desktop through a Virtual Private Network (VPN) specially set up for the server only on administrator machines with Cisco software. The passwords for access were assigned by the provider (web farm ARUBA Spa) directly to the owner of the server administrator (Milan National Cancer Institute). For each maintenance operation that requires access by the web company operators directly to the server, all the passwords for the VPN access are regenerated.
- error handling and traceability. The server log data have been stored since the beginning of the project. However, an applicative access check is provided. In case of malfunction, the server records any errors and keeps them permanently in a database table. Program malfunctions are constantly monitored and whenever they occur a detailed email specifying the kind of error is sent to the administrator. This warning permits prompt assistance for any system failure. Also monitored is the general operation of the server through notifications between the cloud provider and the software administrator.
- database backup. Automatically every day a complete daily backup database copy is generated on the data manager PC. It is also possible to export all the data in a format compatible with the statistical package used (database at https://github.com/ProgettoMeMeMe/MeMeMe).

Our ‘home-made’ CTMS is compliant with 21 CFR part 11 promulgated by the Food and Drug Administration (FDA). In this section of the manuscript we describe the features of our CTMS dealing with “Subpart B—Electronic Records Section 11.10: Controls for closed systems”. A closed system means an environment in which system access is controlled by persons who are responsible for the content of the electronic records on the systems [20]. A randomized controlled clinical trial like Me.Me.Me. generates data that must be managed in a closed environment CTMS, where access and storage security have high priority.

### Room for improvement

This project is ongoing and this software represents the first effort to optimize all the resources available in a no-profit clinical trial. However, there is always room for improvement in a future version of the system. We are expanding the system, introducing an integrated mail server, useful especially for the follow-up of participants; we are working on an automatic way to communicate not only with each participant (i.e. drug expiry date, time to pick up new supply of drugs, etc.), but also with the pharmaceutical company, whenever supplies start to run low. We have implemented an automatic prediction function about drug needs into the software. We are expanding the system with these modules so as to reach an even higher degree of automatic functions.

## Results

The Me.Me.Me. trial is ongoing smoothly, and the recruitment will conclude on August 31, 2018. To date, 1755 volunteers have signed an informed consent and 1054 are currently taking metformin/placebo. Clinical trials usually adopt *patients’ pack*s for drug/placebo doses and drug suppliers are used to the patients’ pack system. Every single participant who has given informed consent had already been assigned a univocal trial code (even an eventual screening failure). Without “*an hoc*” software which plans each medication order, the supplier company would send the full supply of drug for every trial code included in the database (patients’ packs). This is exactly what we avoided in the Me.Me.Me. trial.

We have tested the drug misspending due to a *patients’ packs* system in our trial, according to the real accrual and the drop-out rates, from the first participant’s date of randomization (April 2015). If we had considered a drug supply for the 1755 patients currently recruited, a 36 months of treatment (on average) and a loss at the follow-up of 5%, we would have required 60021 bottles, instead of the 11737 actually dispensed. The difference is due to screening failures before randomization, trend of recruitment, early withdrawals and half-dose supplies for some participants who do not tolerate metformin well or who reduce their metabolic syndrome along the trial. Our on-demand procedure achieved drug savings of 29.5%; this is the result of the following formula: {[(theoretical medication to be ordered according to a *patients’ packs* system)–(number of bottles actually dispensed + estimated number of bottles to conclude the study)]/(theoretical medication to be ordered according to a *patients’ packs* system)}*100. This gives {[(60021)–(11737+30606)] / (60021)}*100 = 29.5%. The estimated number of bottles to conclude the study (30606) takes into account the “state” of participant into the trial, the actual drop-out rate (about 3.2%) and a number that is the average of how many bottles would be needed at full or half dosage to complete the trial.

The total cost of one single bottle of Metformin/Placebo (62 tablets) for our trial is 4.00 Euro, including all the expenses for production, packaging, labeling and warehousing and shipping services. Our drug saving of 29.5% translated into a budget saving of about 71.000 Euro. A part of the money saved (about 15%) was used for paying the software developers and maintenance expenses with a final budget saving of about 60.000 Euro.

## Discussion

Randomized controlled clinical trials are the most powerful study designs to detect associations between intervention and outcome. An information system may offer the means to approach and manage clinical trials in new ways. However, despite the importance of medication management in clinical trials, the scientific literature offers few reports of the implementation of computerized investigational drug services [2,3,4]. The development and implementation of a web-based central computerized system for the Me.Me.Me. trial provides valuable lessons. The basic design reflects what the PI proposed regarding a common framework to support the multiple parts of the clinical trials cycle.

An important aspect of clinical trials is the proper management of data and processes with the least waste of resources. Trials require a specific code for each person who has given signed informed consent. This is essential because GCP require every adverse events occurring during the trial period to be collected and tracked, including the time between signing informed consent and the baseline examinations. Therefore, screening failures at baseline examinations and people who drop out during the trial consume resources. Our CTMS, based on the real ‘state’ of the participant, can minimize waste.

Each trial has different requirements for a system, beyond the basics of enrolling a participant and collecting data, and they often need additional protocol-specific tasks such as those required for the Me.Me.Me. study. A commercial system might not have the flexibility to handle multiple requirements because the architecture of the system is often based on a generic application code. Although this model is very efficient in terms of system validation and maintenance, it therefore loses applicability when a system requires protocol-specific tasks and subsequent steps due to the trial design. On the other, a limit of our system is precisely the lack of an external validity, because of a “*home-grown*” system.

Another limit is that our software is based on Microsoft database technology and language that might not be automatically adaptable to other informatic platforms.

We checked for no-profit software packages already available before starting the Me.Me.Me. trial. For example, the OPENSPECIMEN software for bio-bank improvement and management [21] which may be attractive for universities or big consortiums, but not for independent small research teams. Our software runs the randomization within the same core application, without any support from external tools (such as Interactive Voice Response Systems, IVRS or Interactive Web Response Systems). The randomization process is not integrated in OPENSPECIMEN or in other similar bio-bank management software packages.

The Mayo Clinic circuit created a good CTMS for the no-profit world. From its website, the Mayo Clinic offers to its members three powerful tools: REDCap able to manage studies with low to medium data collection complexity (free); SDMS for studies from medium to complex data management (moderate cost); Medidata Rave for large, complex or multi-site studies (moderate cost) [22]. Collaborative networks as the Mayo Clinic Care Network are very important for improving research improvement but the access procedures for membership are long and complex. Therefore we developed a CTMS for our trial that ensured adequate data and drug management. This ‘home-grown’ system provided the flexibility to address specific issues raised from the trial that are not necessarily applicable to other trials.

## Conclusions

The aim of the Me.Me.Me. trial is to prevent age-related chronic diseases through comprehensive changes in several aspects of the western lifestyle, supported by a chemo-protective agent that acts on the same genetic pathways as the lifestyle factors. We believe that its results will enrich efforts already implemented by the scientific community to clarify the importance of lifestyle for primary prevention and the utility of metformin as a potential chemopreventive agent.

We aimed to demonstrate how an appropriate software can be a powerful resource in developing and managing clinical trials, helping avoid poor treatment allocation, and drug and money waste. Any fixed cost related to the development and maintenance of the software will be depreciated in subsequent years by using our ‘home-made’ system in similar clinical trials. It may prove a powerful resource for developing and conducting future trials.

The MeMeMe software is an open source application. The Me.Me.Me software including the Me.Me.Me. database are fully available for vision, testing and improvement at https://github.com/ProgettoMeMeMe/MeMeMe. This link contains all the instructions for download, installing and using (please view the readme.md file).

We would be glad to share this project with other groups involved in no-profit clinical trials, especially as any such collaboration might lead to further developments and improvements in our ‘home-made’ software, tackling shortcomings and responding to possible future trial needs. We remain available for any clarification or suggestions to help other groups implement this type of database, agreeing with the ‘open science’ way of thinking.

## Clinical relevance statement

Clinical trial investigators and their team invest vast amounts of resources and energy in conducting research studies and often face daily challenges with data management, project management, and data quality control. An information system “*ad hoc*” may offer to the means to approach and manage clinical trials in a new way. A software can be a powerful resource for developing and managing clinical trials, to avoid poor treatment allocation, and drug and money waste.

## Supporting information

S1 File MeMeMe_PROTOCOL. (DOCX)

S2 File MeMeMe_Checklist. (DOC)

S1 Fig Trial design. Red symbols refer to participants lost from the study.
(TIF)

S2 Fig The encrypted screen. The first column shows the bottle number, the second column shows the randomization block, and the third column shows the encrypted code about metformin/placebo treatment.
(TIF)
