# Supplementary figures and images for: A management system for randomized clinical trials: A novel way to supply medication
Source: PLoS One. 2019 Feb 22;14(2):e0212475. doi: 10.1371/journal.pone.0212475 (PMC6386477; doi:10.1371/journal.pone.0212475)

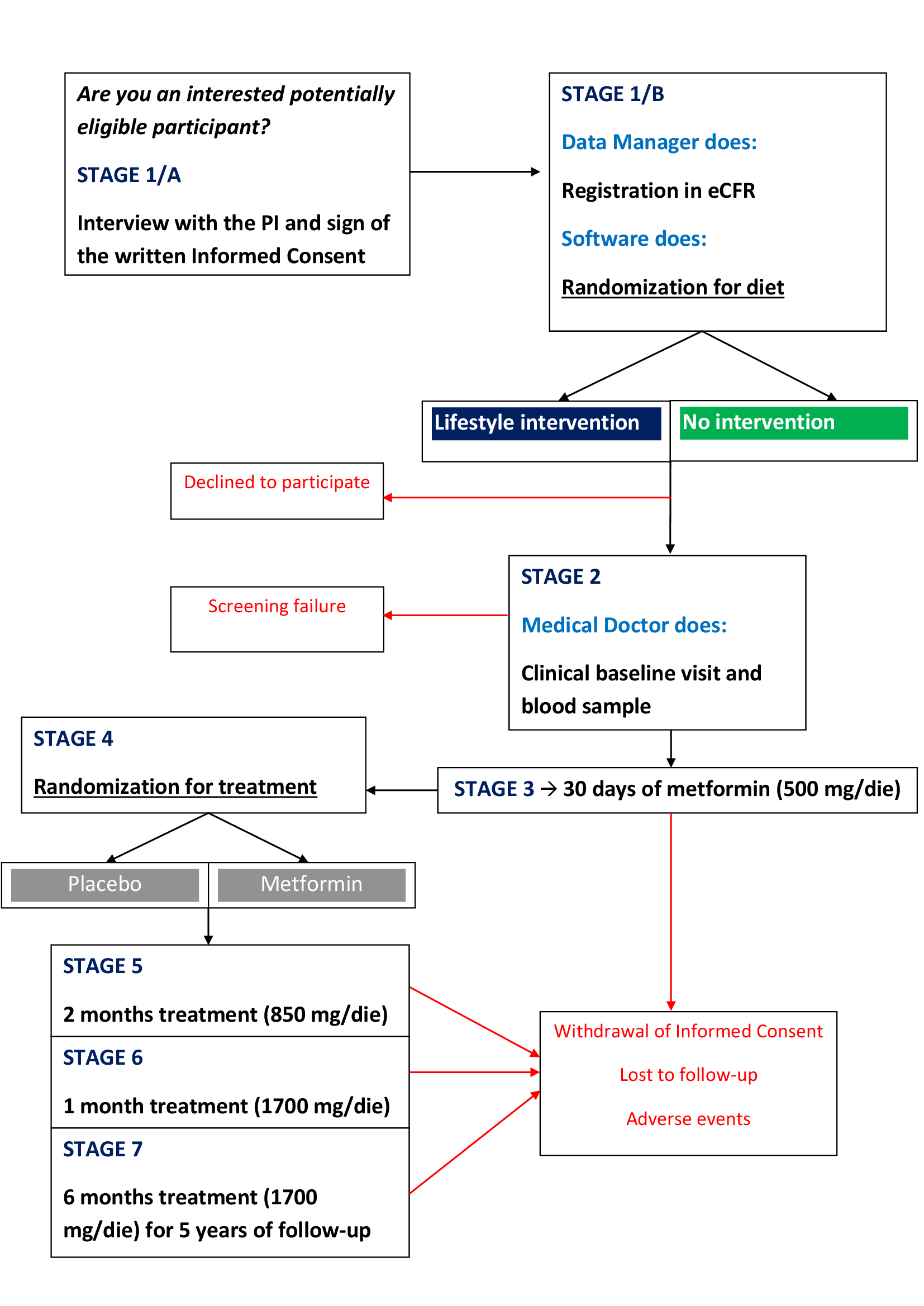

Supplement: S1 Fig — Red symbols refer to participants lost from the study. (TIF) [file pone.0212475.s003.tif]

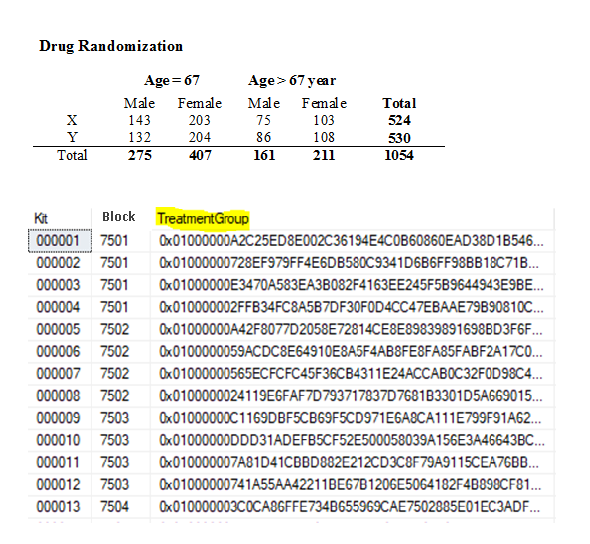

Supplement: S2 Fig — The first column shows the bottle number, the second column shows the randomization block, and the third column shows the encrypted code about metformin/placebo treatment. (TIF) [file pone.0212475.s004.tif]
